# Serum sialyl transferase levels in patients with metastatic breast cancer treated by chemotherapy.

**DOI:** 10.1038/bjc.1982.185

**Published:** 1982-08

**Authors:** J. F. Stewart, R. D. Rubens, S. Hoare, R. D. Bulbrook, D. Kessel

## Abstract

Serum sialyl transferase (SST) was measured in 49 female patients with advanced breast cancer and 60 female controls. The mean SST level was significantly raised in patients with advanced breast cancer. There was no correlation between specific sites, or numbers of sites of metastatic disease and SST levels. The patients with advanced breast cancer were all treated with chemotherapy; in 13/21 responders there was a significant fall in SST and in 2 responders a significant rise in SST. The 6 patients who died after one course of chemotherapy had a significantly higher mean SST than those surviving longer. SST appears to lack sufficient specificity to be of practical value as a marker of response in patients with breast cancer treated by chemotherapy, though constant measurement of changes in SST may be of use in monitoring drug response. Raised SST at the commencement of chemotherapy may signify poor prognosis.


*Br. J. Cancer* (1982) **46**, 208

### SERUM SIALYL TRANSFERASE LEVELS IN PATIENTS WITH METASTATIC BREAST CANCER TREATED BY CHEMOTHERAPY

J. F. STEWART*, R. D. RUBENS*, S. HOARE†, R. D. BULBROOK† AND D. KESSEL‡

*From the *Imperial Cancer Research Fund, Breast Cancer Unit, Guy's Hospital, London SE1 9RT, the †Department of Clinical Endocrinology, Imperial Cancer Research Fund, Lincoln's Inn Fields, London WC2A 3PX, and the ‡Department of Oncology/Pharmacology, Wayne State University, Detroit, Michigan* 48201, *U.S.A.*

Received 12 November 1981 Accepted 2 August 1982

**Summary.—Serum sialyl transferase (SST) was measured in 49 female patients with advanced breast cancer and 60 female controls. The mean SST level was significantly raised in patients with advanced breast cancer. There was no correlation between specific sites, or numbers of sites of metastatic disease and SST levels. The patients with advanced breast cancer were all treated with chemotherapy; in 13/21 responders there was a significant fall in SST and in 2 responders a significant rise in SST. The 6 patients who died after one course of chemotherapy had a significantly higher mean SST than those surviving longer. SST appears to lack sufficient specificity to be of practical value as a marker of response in patients with breast cancer treated by chemotherapy, though constant measurement of changes in SST may be of use in monitoring drug response. Raised SST at the commencement of chemotherapy may signify poor prognosis.**

THE GLYCOSYL TRANSFERASES are a group of enzymes involved in glycoprotein synthesis; they catalyse addition of specific monosaccharides to the growing glycoprotein molecule. There may be alterations in the composition of the various cell-surface glycoproteins in malignancy (Van Beek *et al.*, 1973) and, associated with this, elevations in the different glycosyl transferases found in the sera of patients with neoplastic disease. Plasma fucosyl transferase (Khilanani *et al.*, 1978) and galactosyl transferase levels (Paone *et al.*, 1978) have been shown to be increased in some malignancies. More particularly, serum and plasma sialyl transferase has been found to be raised in patients with breast (Ganzinger & Moser, 1979) and other cancers (Dao *et al.*, 1980; Ganzinger & Deutsch, 1980). Levels of this enzyme are reported to reflect the extent of metastatic disease and response to treatment.

We have measured serum sialyl transferase (SST) levels in patients with advanced breast cancer and, as part of a clinical trial comparing two different chemotherapy regimens, compared serial SST levels with the clinical response to treatment.

### METHODS AND MATERIALS

*Serum sialyl transferase assay.*—Cytidine 5′-monophosphate sialic acid, sialic-4 ($^{14}C$) specific activity 1·68 mCi/mmol was purchased from New England Nuclear Corp. Ltd. The desialated acceptor was prepared from calf thymus fetuin (Sigma) by removal of the terminal sialic acid residue.

Analytical-grade anion exchange resin, Ag-2-X8 (100–200 mesh size) in the chloride form (Bio-Rad Laboratories) was converted into the hydroxide form by treatment with 1M NaOH, followed by repeated washings with distilled water until pH 7 was achieved.

The scintillation fluid Aquasol was purchased from New England Nuclear Corp. Ltd. All samples were counted in a Packard

PLD liquid scintillation counter in plastic vials obtained from Packard.

*Preparation of desialated fetuin.*—Calf thymus fetuin 1 g was dissolved in 25 ml of distilled water and the terminal sialic acid residues removed by the action of 1·25 ml 1M $H_2SO_4$ at 80°C for 1 h. The solution was chilled, neutralized to pH 7 with 1M NaOH, and dialysed for 4 h against a 0·5% NaCl solution, before finally dialysing overnight against distilled water. This dialysed solution was freeze-dried to yield a white powder of desialated fetuin.

*Sialyl transferase assay.* — The complete assay system contained 25 $\mu$l serum, 3 $\mu$l 1M Hepes buffer (pH 7·0), 10 $\mu$l 0·1M $MgCl_2$, 10 $\mu$l desialated fetuin (20 mg/ml), 5 $\mu$l (1 nmol) Cytidine 5′-monophosphate ($^{14}C$) sialic acid, and 60 $\mu$l distilled water. This was incubated for 30 min at 37°C. The product, desialated fetuin terminally labelled with ($^{14}C$) sialic acid, was isolated using ion-exchange chromatography and counted in a liquid-scintillation counter. The mean of two replicate samples was used in this analysis. The interassay variation of 15 determinations and the intra assay variation of 40 determinations were both ~5%.

*Patients.*—Forty-nine patients with progressive metastatic breast cancer, treated previously by endocrine therapy (ovarian ablation, androgens, oestrogens or anti-oestrogens ± subsequent hypophysectomy) but not prior chemotherapy were treated with Adriamycin (Adr, 70 mg/m$^2$) i.v. (60 mg/m$^2$ for patients >60 years) on day 1 of a 3-weekly cycle for 8 cources, followed by a regimen of cyclophosphamide (100 mg/m$^2$ p.o., Days 1–14) + methotrexate (30 mg/m$^2$ i.v., Days 1 and 8) + 5-fluorouracil (600 mg/m$^2$ i.v., Days 1 and 8) 4× weekly until relapse. They were allocated randomly to receive either no other treatment or vincristine (1·4 mg/m$^2$) on Days 1 and 8 during treatment with Adr. Before chemotherapy commenced, patients were assessed clinically and with bone scans (including radiographs of areas of increased uptake), chest radiograph, full blood count, biochemical screen and liver scan. A selection of baseline lesions for serial assessment was obtained. Before each i.v. injection, a full blood profile (including platelets) was done and before each cycle of chemotherapy patients were examined and repeat assessment of baseline lesions made. Every 3 months patients had repeat radiographs and liver scans if clinically indicated. Response to treatment was assessed by UICC criteria (Hayward *et al.*, 1977).

*Controls.*—Sera were collected from female volunteers aged 35+ years as part of a larger study of the aetiology of breast cancer (Farewell *et al.*, 1978). All volunteers had a clinical examination and mammography to exclude breast pathology.

*Serum collection.*—Blood samples from patients and controls were collected, allowed to stand for 1 h, centrifuged at 800 *g* and the resultant serum was stored at −20°C until assayed. Serum was collected immediately before each cycle of chemotherapy. Sera from controls were assayed within 1 month of collection, whilst sera from patients were stored for periods of 1 to 24 months before assay. There were no differences in baseline enzyme activity between patients whose sera was assayed within 1 month of collection (n = 13), from 1 to 6 months of collection (n = 16) or for longer than this period (n = 20). In addition, in a sample assayed on 40 occasions over 2 years, the coefficient of variation was 5%. These results suggest that storage at −20°C for up to 2 years does not significantly alter serum enzyme activity.

*Statistical methods.*—Comparisons of group mean values were tested for significance by using the *t* test. The significance of differences between binary variables was calculated by the $\chi^2$ test for 1 degree of freedom.

### RESULTS

### *Response to chemotherapy*

Twelve of 25 (48%) patients responded to Adr alone and 3/25 (12%) had stable disease, while 9/14 (38%) responded to the combination of Adr and vincristine and 6/24 (25%) had stable disease.

### *Baseline values*

The mean baseline serum sialyl transferase (SST) level in patients with advanced breast cancer was higher than in controls (Table I). In addition, mean baseline levels are shown for each category of response to chemotherapy. The 6 patients who died after the first course of chemotherapy had a mean SST of 406 ± 76

TABLE I.—*Response categories and mean baseline serum sialyl transferase (SST) estimation*

| | SST (ct/min/ mg protein/h) | |
|---|---|---|
| Controls (60) | *258 ± 41 | $P < 0.01$ |
| All patients (49) | 340 ± 112 | |
| Responders (21) | 338 ± 102 | |
| Progressive disease (19) | 342 ± 84 | |
| No change (9) | 299 ± 213 | |

* Mean SST ± s.d. Figures in parentheses refer to numbers of patients.

TABLE III.—*Baseline SST and sites of disease at commencement of chemotherapy. Figures in brackets refer to number of patients*

| Sites of disease | SST ± s.d. (ct/min/mg protein/h) |
|---|---|
| Soft tissue only (11) | 338 ± 176 |
| Skeletal (30) | 332 ± 88 |
| Hepatic (5) | 345 ± 79 |
| Visceral (liver and lung) (14) | 359 ± 88 |
| Non-visceral (35) | 332 ± 119 |
| One site only (18) | 339 ± 139 |
| More than one site (31) | 340 ± 91 |

ct/min/mg protein/h, compared to a mean SST of 331 ± 115 for other chemotherapy patients ($P < 0.05$). The SST before chemotherapy, above or below an arbitrarily chosen value of 350 ct/min was of some use in predicting an individual response category (Table II). Twenty-two of 30 patients with a pretreatment SST of < 350 ct/min responded to or had stable disease with chemotherapy, compared to only 8/19 with a higher baseline level ($\chi^2$ 4·78, $P < 0.05$).

Table III shows the mean SST according to individual disease sites and numbers of sites involved at the commencement of chemotherapy. There was no clear relationship between SST levels and any site, or numbers of sites, of disease involvement.

*Serial values*

Measurement of SST at any given time (*e.g.* 3–6 weeks) after the initiation of treatment had no prognostic value. However, if the pretreatment value for a particular patient was used as a reference point, then changes in SST were weakly related to response. If the SST level was above the baseline 6 weeks after treatment, the response + no-change rate was 8/16 patients: if SST levels had fallen the response + no-change rate was 18/19 patients ($\chi^7 = 9.1$, $P < 0.01$). At 6 weeks, clinical response had been noted in only 6/21 (29%) of patients who eventually responded to chemotherapy.

If the SST levels were below the pretreatment value at both 3 and 6 weeks after treatment, the response + no-change rate was 12/12 patients. If SST was raised both times, the response + no-change rate was 6/11 patients ($\chi^2 = 6.96$, $P < 0.05$). As no data were available on the changes in serial SST with time in patients with untreated metastatic disease, a significant change in SST was arbitrarily regarded as > s.d. of controls (*viz.* > 41 ct/min/mg protein/h).

Table IV shows the relationship between the lowest value of SST and response category. In 13/21 responders there was a fall in SST in remission, and in 8/21 responders this was a fall of > 2 s.d. (*viz.* > 82 ct/min/mg protein/h), whilst in the non-responders there was a fall in 4/13. and in only 1/13 was this fall > 2 s.d. These differences are not significant.

TABLE II.—*Baseline SST estimations (ct/min/mg protein/h) and response categories*

| | Response category | | | |
|---|---|---|---|---|
| Baseline SST | Responders | No change | (Total responders + no change) | Progressive disease |
| ⩽ 350 | 14 | 8 | 22 (73%) | 8 (27%) |
| > 350 | 7 | 1 | 8 (42%) | 11 (58%) |
| Total | 21 | 9 | 30 | 19 |

TABLE IV.—*Lowest SST (ct/min/mg protein/h) relative to baseline, and response category*

| Lowest SST relative to baseline | Response category | | |
|---|---|---|---|
| | Response | No change | Progressive disease |
| $- > 82$ | 8 | 1 | 1 |
| $- > 41$ | 5 | 3 | 3 |
| No change ($\pm 41$) | 6 | 3 | 7 |
| $+ > 41$ | 2 | 1 | 2 |
| $+ > 82$ | 0 | 1 | 0 |

There was a significant difference ($P <$ 0·025) in the lowest serial value of SST in responders ($252 \pm 94$ ct/min/mg protein/h) compared to patients with progressive disease ($321 \pm 94$ ct/min/mg protein/h). In patients responding to chemotherapy, there was no consistent relationship between lowest SST and time from commencement of chemotherapy, lowest values being found from 3 to 55 weeks after commencement of chemotherapy. Eleven responders and 3 patients in the no-change category have been followed until relapse. Table V shows the difference between the lowest SST in remission and at relapse. There was a significant difference ($P < 0·05$) between the lowest SST in remission ($232 \pm 97$) and the level at relapse ($304 \pm 100$). Since there were often some months between the time of the lowest SST and the time of relapse, a rise of SST in remission was of little value in predicting onset of relapse.

TABLE V.—*Difference between lowest SST in remission and value at relapse*

| Difference in SST (ct/min) | Response category | |
|---|---|---|
| | Responder | No change |
| No change $\pm < 41$ | 5 | 2 |
| $+ > 41$ | 5 | 1 |
| $- > 41$ | 1 | 0 |

### DISCUSSION

High SST levels in advanced breast cancer has been reported by several groups (Ganzinger & Moser, 1979; Dao *et al.*, 1980; Kessel & Allen, 1975), and our results confirm these findings. Unlike other reports (Dao *et al.*, 1980; Henderson & Kessel, 1977), there was no clear relationship between SST levels and sites or bulk of metastic disease.

It has been shown that surgical removal of tumour tissue will lead to a fall in SST, and similarly, after ablative endocrine therapy, objective regression of disease is accompanied by a fall in SST (Dao *et al.*, 1980). In this study it was found that in 11 patients with advanced breast cancer treated with chemotherapy, significant falls in SST occurred in all 3 responders and significant rises in all 4 patients with progressive disease. Similar results have been shown by others (Ganzinger & Moser, 1979). Our results also show a fall in SST is often associated with successful chemotherapy, and that a fall may precede clinical evidence of response. One contradictory finding that emerges from this study is that although no relationship between SST and bulk of disease has been demonstrated, changes in level do reflect response to treatment and, presumably, change in tumour burden. All patients in this study had extensive disease, even if only one site was involved, and there is some evidence from animal models that there is a direct relationship between sialyl transferase activity and tumour burden, but only up to a certain threshold, above which increases in tumour burden are not reflected by increases in SST activity (Evans *et al.*, 1980). It is possible that the existence of a similar phenomenon in human breast cancer may explain this apparent discrepancy.

Constant and accurate measurement of changes in SST may be of some use in monitoring response to chemotherapy, but both false-negative results (a rise in SST associated with a response to chemotherapy) and false-positive results (a fall in SST, associated with disease progression) occur. In view of this lack of specificity we are investigating the possibility that a tumour-associated isoenzyme of sialyl transferase may be a more useful marker for the follow-up of patients with breast cancer (Kessel *et al.*, 1981).

### REFERENCES

DAO, T., IP, C. & PATEL, J. (1980) Serum sialyltransferase and 5′-nucleotidase as reliable biomarkers in women with breast cancer. *J. Natl Cancer Inst.*, **65**, 529.

EVANS, I., HILF, R., MURPHY, M. & BOSMANN, H. (1980) Correlation of serum, tumour and liver serum glycoprotein: n-Acetylneuraminic acid transferase activity with growth of the R3230 AC mammary tumour in rats and relationship of the serum activity to tumour burden. *Cancer Res.*, **40**, 3103.

FAREWELL, V., BULBROOK, R. & HAYWARD, J. (1978) Risk factors in breast cancer: A prospective study in the Island of Guernsey. In *Cancer Campaign*, Vol. I. *Early Diagnosis of Breast Cancer—Methods and Results* (Ed. Grundmann & Beck). Stuttgart: Gustaf Fischer p.43.

GANZINGER, U. & MOSER, K. (1979) Sialyltransferase activity: A serum marker in the follow-up of cancer patients. *Recent Results Cancer Res.*, **67**, 50.

GANZINGER, U. & DEUTSCH, E. (1980) Serum sialyltransferase levels as a parameter in the diagnosis and follow-up of gastrointestinal tumours. *Cancer Res.*, **40**, 1300.

HAYWARD, J., RUBENS, R., CARBONE, P., HEUSON, J., KUMAOKA, S. & SEGALOFF, A. (1977) Assessment of response to therapy in advanced breast cancer. *Br. J. Cancer*, **35**, 292.

HENDERSON, M. & KESSEL, D. (1977) Alterations in plasma sialyltransferase levels in patients with neoplastic disease. *Cancer*, **38**, 1129.

KESSEL, D. & ALLEN, J. (1975) Elevated plasma sialyltransferase in the cancer patient. *Cancer Res.*, **35**, 670.

KESSEL, D., CHOU, T. & COOMBES, R. (1981) Studies on sialyl-transferase isoenzymes in plasma of patients with breast cancer. *Eur. J. Cancer*, **17**, 1035.

KHILANANI, P., CHOU, T., RATANATHARATHORN, N. V. & KESSEL, D. (1978) Evaluation of two plasma fucosyltransferases as marker enzymes in non-Hodgkin's lymphoma. *Cancer* **41**, 701.

PAONE, J., WAALKES, T., ROBINSON BAKER, R. & SHARPER, J. (1978) Positive correlation of serum uridine diphosphate–galactosyltransferase levels with breast carcinoma. *Surg. Forum.* **29**, 159.

VAN BEEK, W., SMETS, L. & EMMELOT, P. (1973) Increased sialic acid density in surface glycoprotein of transformed and malignant cells: A general phenomenon. *Cancer Res.*, **33**, 2913.


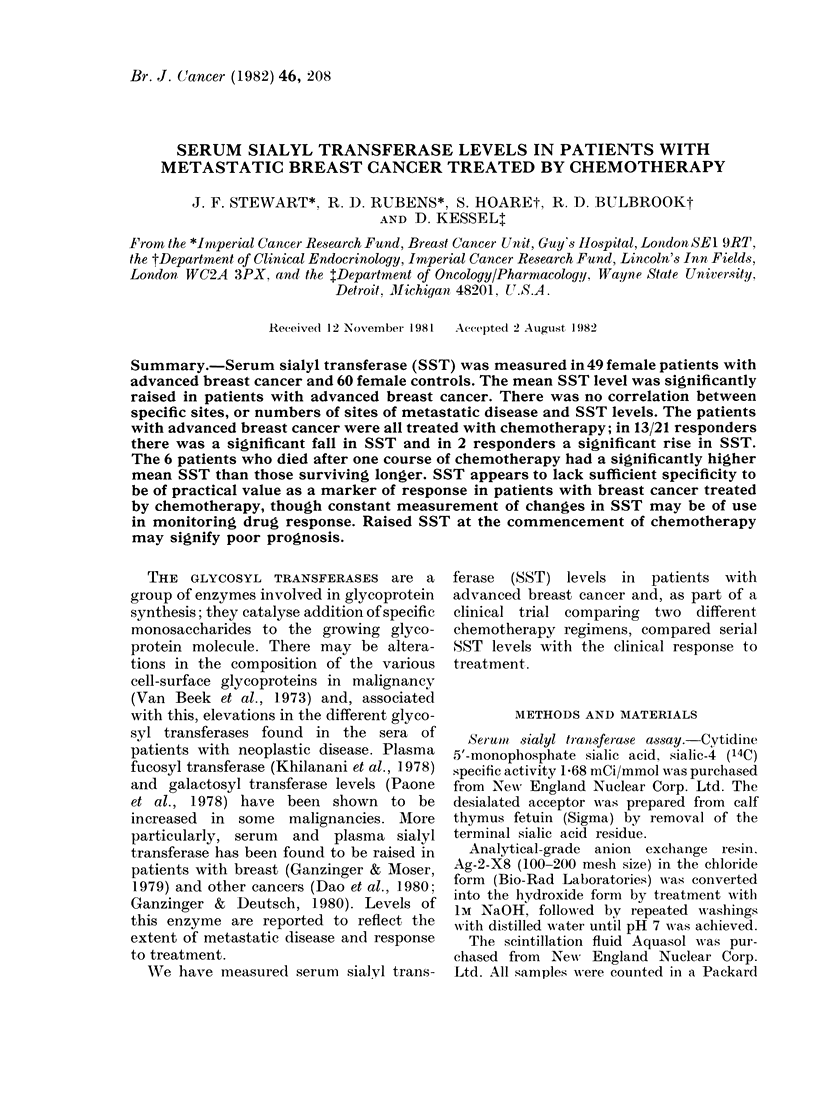

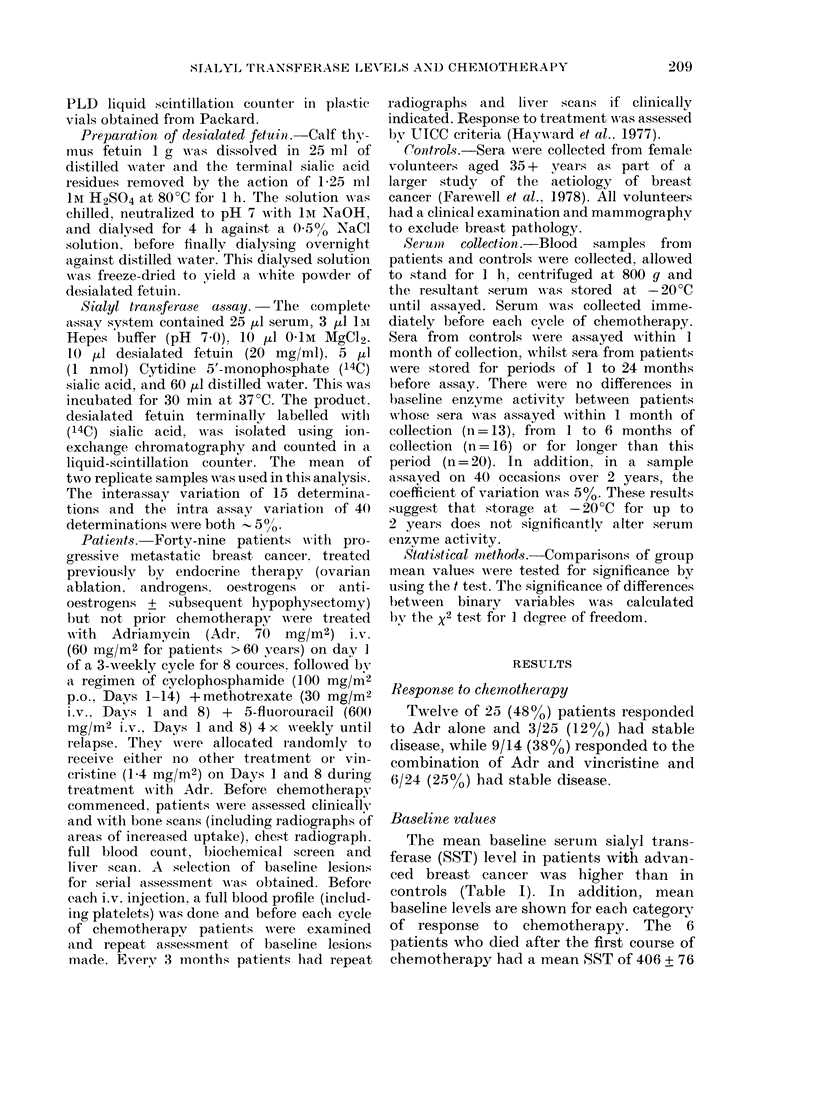

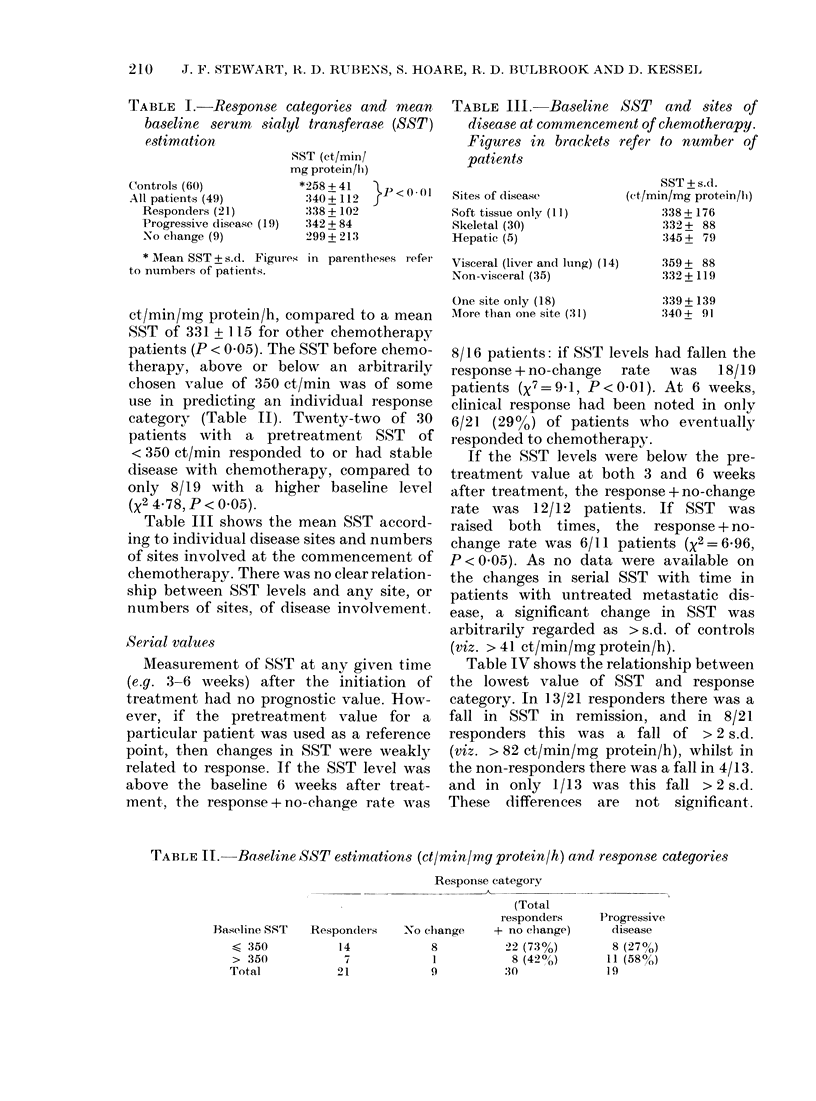

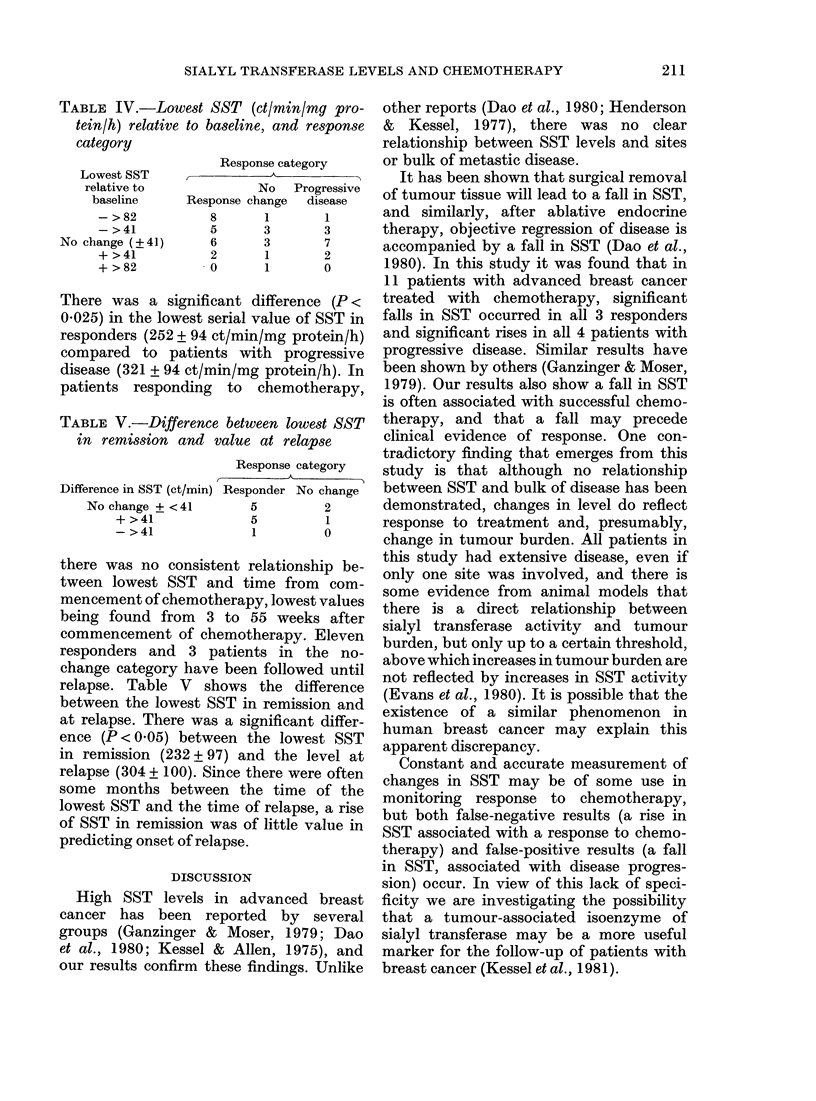

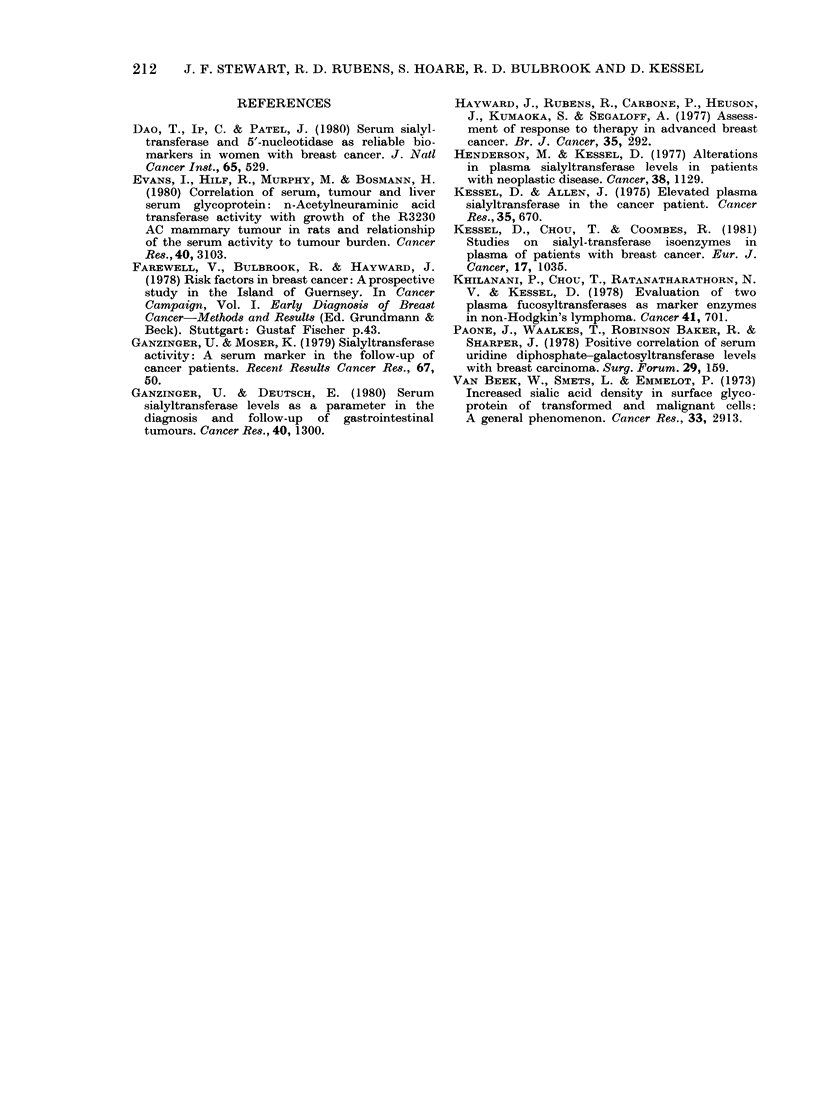


## References

[OCR_00515] Dao T. L., Ip C., Patel J. (1980). Serum sialyltransferase and 5'-nucleotidase as reliable biomarkers in women with breast cancer.. J Natl Cancer Inst.

[OCR_00521] Evans I. M., Hilf R., Murphy M., Bosmann H. B. (1980). Correlation of serum, tumor, and liver serum glycoprotein: N-acetylneuraminic acid transferase activity with growth of the R3230AC mammary tumor in rats and relationship of the serum activity to tumor burden.. Cancer Res.

[OCR_00544] Ganzinger U., Deutsch E. (1980). Serum sialyltransferase levels as a parameter in the diagnosis and follow-up of gastrointestinal tumors.. Cancer Res.

[OCR_00538] Ganzinger U., Moser K. (1979). Sialyl transferase activity: a serum enzyme marker in the follow-up of cancer patients.. Recent Results Cancer Res.

[OCR_00550] Hayward J. L., Carbone P. P., Heusen J. C., Kumaoka S., Segaloff A., Rubens R. D. (1977). Assessment of response to therapy in advanced breast cancer.. Br J Cancer.

[OCR_00556] Henderson M., Kessel D. (1977). Alterations in plasma sialyltransferase levels in patients with neoplastic disease.. Cancer.

[OCR_00566] Kessel D. H., Chou T. H., Coombes R. C. (1981). Studies on sialyltransferase isoenzymes in plasma of patients with breast cancer.. Eur J Cancer Clin Oncol.

[OCR_00561] Kessel D., Allen J. (1975). Elevated plasma sialyltransferase in the cancer patient.. Cancer Res.

[OCR_00572] Khilanani P., Chou T. H., Ratanatharathorn V., Kessel D. (1978). Evaluation of two plasma fucosyltransferases as marker enzymes in non-Hodgkin's lymphoma.. Cancer.

[OCR_00584] van Beek W. P., Smets L. A., Emmelot P. (1973). Increased sialic acid density in surface glycoprotein of transformed and malignant cells--a general phenomenon?. Cancer Res.

